# Paw Skin as a Translational Model for Investigating Fibrotic and Inflammatory Wound Healing Defects in Recessive Dystrophic Epidermolysis Bullosa

**DOI:** 10.3390/ijms26094281

**Published:** 2025-04-30

**Authors:** Cristian De Gregorio, Giselle Ramos-Gonzalez, Bernardo Morales-Catalán, Fernando Ezquer, Marcelo Ezquer

**Affiliations:** 1 Centro de Medicina Regenerativa, Facultad de Medicina, Clínica Alemana-Universidad del Desarrollo, Santiago 7610658, Chile; giselle.ramos@udd.cl (G.R.-G.); eezquer@udd.cl (F.E.); 2 Centro de Anatomía Patológica, Biología Molecular y Genética, ATRYS, Santiago 7510249, Chile; labdrmorales@gmail.com

**Keywords:** RDEB, skin fibrosis, inflammation, wound healing, sensory nerve alteration, paw skin model

## Abstract

Recessive dystrophic epidermolysis bullosa (RDEB) is a severe genetic disease caused by *COL7A1* mutations. It leads to skin fragility, chronic inflammation, and impaired wound healing. The condition often results in fibrotic scarring, pseudosyndactyly, and cutaneous squamous cell carcinoma (SCC). However, current animal models fail to fully replicate chronic RDEB wounds. In this study, we used Collagen VII-hypomorphic mice (*Col7a1^flNeo/flNeo^*) and created full-thickness wounds on their paw skin, an area prone to fibrosis due to mechanical stress. We analyzed the healing process using histology, immunofluorescence, and electron microscopy. The RDEB mice showed delayed wound closure, increased inflammation, and poor granulation tissue formation. At 30 days post-injury, we observed persistent fibrosis, with elevated levels of Collagen I, α-SMA+ myofibroblasts, and tenascin-C. These mice also had fewer intraepidermal nerve fibers, which may help explain the neuropathic pain associated with RDEB. Our model reproduces the main features of chronic RDEB wounds. It offers a useful tool for evaluating therapies aimed at reducing inflammation, fibrosis, and tumor risk in these patients.

## 1. Introduction

The skin is a complex, multi-layered organ that functions as a biological and physical barrier, capable of self-renewal and regeneration after injury [1,2]. The wound healing process involves a highly coordinated series of events, including hemostasis, inflammation, cell proliferation, migration, and extracellular matrix (ECM) remodeling [3]. In normal conditions, inflammation resolves itself in a timely manner, allowing the transition into the proliferative phase. However, in recessive dystrophic epidermolysis bullosa (RDEB), a severe genetic disorder caused by mutations in the *COL7A1* gene [4], which encodes type VII collagen (C7), these processes are profoundly disrupted. RDEB is characterized by extreme skin fragility, chronic inflammation, impaired wound healing, and a high risk of fibrotic scarring, pseudosyndactyly, and cutaneous squamous cell carcinoma (SCC) [5,6].

Epidermolysis bullosa (EB) as a whole is a heterogeneous group of hereditary disorders caused by mutations affecting keratin filaments, desmosomes, hemidesmosomes, and adhesion proteins, leading to the disruption of the dermal-epidermal junction (DEJ) [7].

Despite increasing knowledge of the molecular defects underlying RDEB, effective therapeutic development remains hampered by the lack of accurate preclinical models that faithfully recapitulate the chronic, fibrotic, and inflammatory features of this human disease. Current murine models, including the hypomorphic *Col7a1^flNeo/flNeo^* mouse model (C7-hypomorphic mice), which expresses ~10% of wild-type C7, predominantly use back skin wounds, which differ from human wounds in their healing dynamics and tissue architecture [8,9]. Moreover, the back skin exhibits lower susceptibility to fibrosis compared to high-friction anatomical sites, such as the paws [8].

Given that clinical studies have reported that over 90% of chronic wounds and ~95% of SCC cases in RDEB patients occur in extremities [10], there is a critical need for preclinical models that better mimic the pathological microenvironment of chronic wounds in these areas.

A key factor in wound healing is the differentiation of fibroblasts into myofibroblasts, driven mainly by transforming growth factor beta 1 (TGF-β1), which regulates α-SMA expression and the production of fibrotic ECM components like Collagen I and tenascin-C [11,12,13]. In physiological conditions, myofibroblasts undergo apoptosis after completing their function; however, in RDEB, persistent myofibroblast activation leads to excessive ECM accumulation, increased tissue stiffness, and fibrosis [14]. This triggers a pathological loop perpetuating fibrosis and impairing proper wound healing [15].

Therefore, the objective of this study is to characterize the wound healing impairments in C7-hypomorphic mice by employing a paw skin model, which is exposed to mechanical stress and is prone to fibrosis. This approach aims to better capture the interplay between the chronic inflammation, fibrosis, and sensory nerve alterations seen in human RDEB, offering a more physiologically relevant platform for studying disease mechanisms and evaluating regenerative therapies.

## 2. Results

### 2.1. C7-Hypomorphic Mice Exhibit Growth Impairments but No Increased Mortality at Three Months of Age

Previous studies have shown that C7-hypomorphic mice present a reduced lifespan and growth retardation, along with characteristic RDEB manifestations, including skin fragility, fibrosis, mitten deformities, and pseudosyndactyly [8,9,16,17]. In our study, we confirmed that C7-hypomorphic mice displayed a 50% lower body weight compared to control littermates (Supplementary Figure S1a). Interestingly, hind paw size was similar between the groups at 13 weeks of age (Supplementary Figure S1b), suggesting that nutritional deficiencies, rather than intrinsic developmental defects, may be responsible for growth impairments, as previously reported in human RDEB patients [18,19].

Contrary to previous reports, our study found no significant increase in mortality at 13 weeks of age in C7-hypomorphic mice (Supplementary Figure S1c). We attribute this finding to optimized supportive care, including nutritional supplementation and soft bedding, which likely enhanced survival by preventing severe malnutrition and spontaneous injuries.

### 2.2. Delayed Wound Healing in C7-Hypomorphic Mouse Paw Skin

Previous studies by Nyström et al. demonstrated that C7 reduction is associated with delayed wound healing and impaired re-epithelialization in the back skin of RDEB mice [9]. Although the splinted wound model in the back skin is widely used in rodents to analyze re-epithelialization [20], its application in C7-hypomorphic mice is particularly challenging due to their skin fragility and reduced body size.

It is important to note that back skin in mice generally exhibits lower levels—or even an absence—of inflammation and fibrosis compared to high-friction areas such as the paws [8]. Indeed, RDEB mouse paw skin is particularly prone to fibrotic damage, leading to the development of digital malformations and pseudosyndactyly as early as 2–3 months of age [8,9]. These pathological alterations closely resemble those observed in patients with severe RDEB, further highlighting the clinical relevance of this mouse model for understanding disease mechanisms.

**Figure 1:**
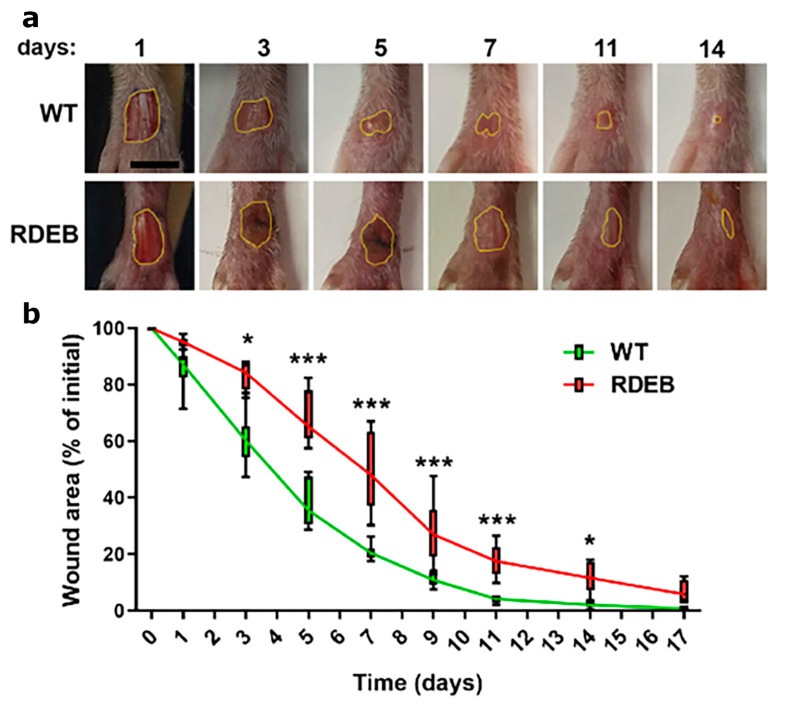
Delayed wound closure in RDEB mice using a full-thickness paw skin wound model. (**a**) Representative digital images of the hind paws of mice subjected to excisional full-thickness wounding. The yellow line delimits the wound area. Scale bar = 2.5 mm. (**b**) Wound closure analysis. The wound area is expressed as a percentage relative to the initial wound size. Data are presented as mean ± SEM (*n* = 8; 4 males and 4 females per genotype). Asterisks indicate significant differences (* *p* < 0.05; *** *p* < 0.001) determined by a two-tailed Mann–Whitney test.

To investigate wound healing in a fibrotic and high-friction environment, we performed a full-thickness skin excision on the dorsal surface of the hind paws of both C7-hypomorphic and WT littermates and analyzed wound closure kinetics. Our results showed that C7-hypomorphic mice exhibited a significant delay in wound healing compared to their WT littermates, with larger wound areas observed from day 3 post-injury (Figure 1a,b). The most pronounced differences in wound size occurred between days 3 and 7, a critical period when wounds are particularly susceptible to pathogen contamination and chronicity. After day 14, wound closure kinetics began to converge between the groups, consistent with previous observations in back skin wounds of RDEB mice [9].

These findings suggest that hind paw skin represents a physiologically relevant model for studying delayed wound healing in RDEB, providing valuable insights into fibrotic wound pathology and potential therapeutic strategies.

**Figure 2:**
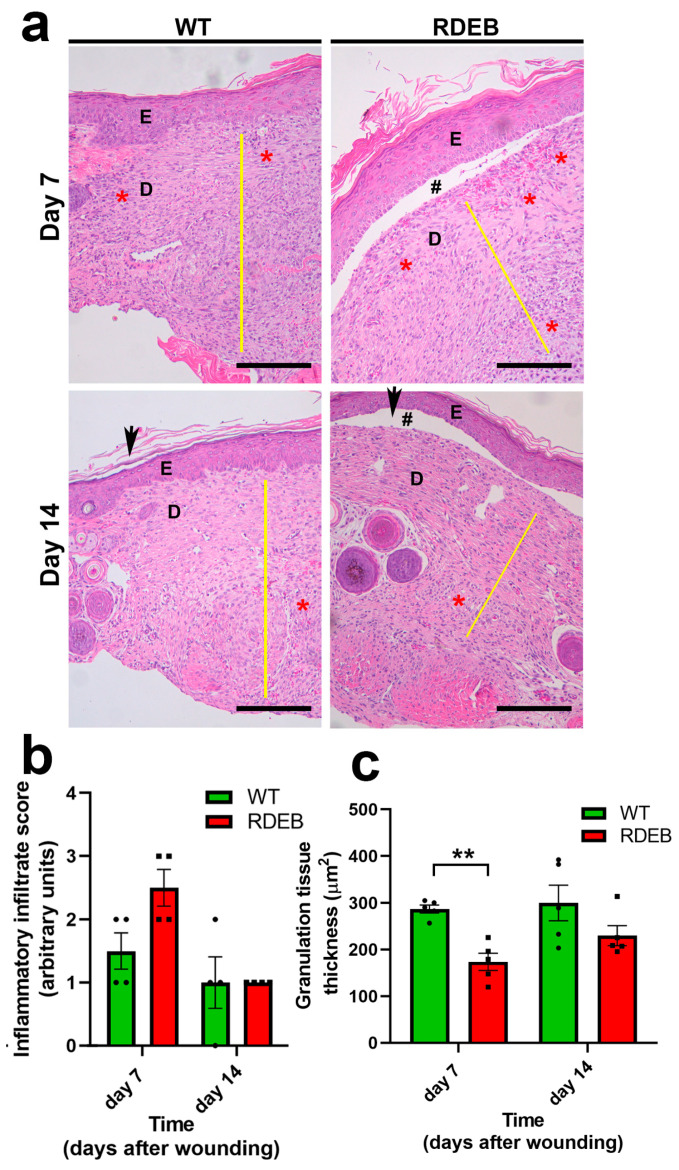
Histological impairments in RDEB mice associated with delayed wound closure in paw skin. (**a**) Representative hematoxylin and eosin (H&E)-stained images of skin samples from WT and RDEB mice at days 7 and 14 post-injury. Yellow lines indicate the thickness of the granulation tissue, while red asterisks mark regions of inflammatory cell infiltration. E: epidermis; D: dermis; # indicates skin blistering; arrows indicate the wound border. Scale bar = 100 μm. (**b**,**c**) Quantitative analysis of the inflammatory infiltrate score (**b**) and granulation tissue thickness (**c**). Data are expressed as mean ± SEM (*n* = 4 per genotype). Asterisks denote statistically significant differences (** *p* < 0.01) determined by a two-tailed Mann–Whitney test.

To further characterize the mechanisms that may be associated with delayed wound closure, we conducted a histopathological analysis 7 and 14 days post-wound induction. Through hematoxylin and eosin (H&E) staining, we analyzed the total number of inflammatory cells and the formation of granulation tissue. The data indicate a slight increase, although not significant, in inflammatory cell infiltrate (Figure 2a,b). However, a decrease in the thickness of the granular tissue can be observed in the RDEB mice on day 7 (Figure 2a,c), which could be associated with an impaired regenerative capacity compared to WT animals.

**Figure 3:**
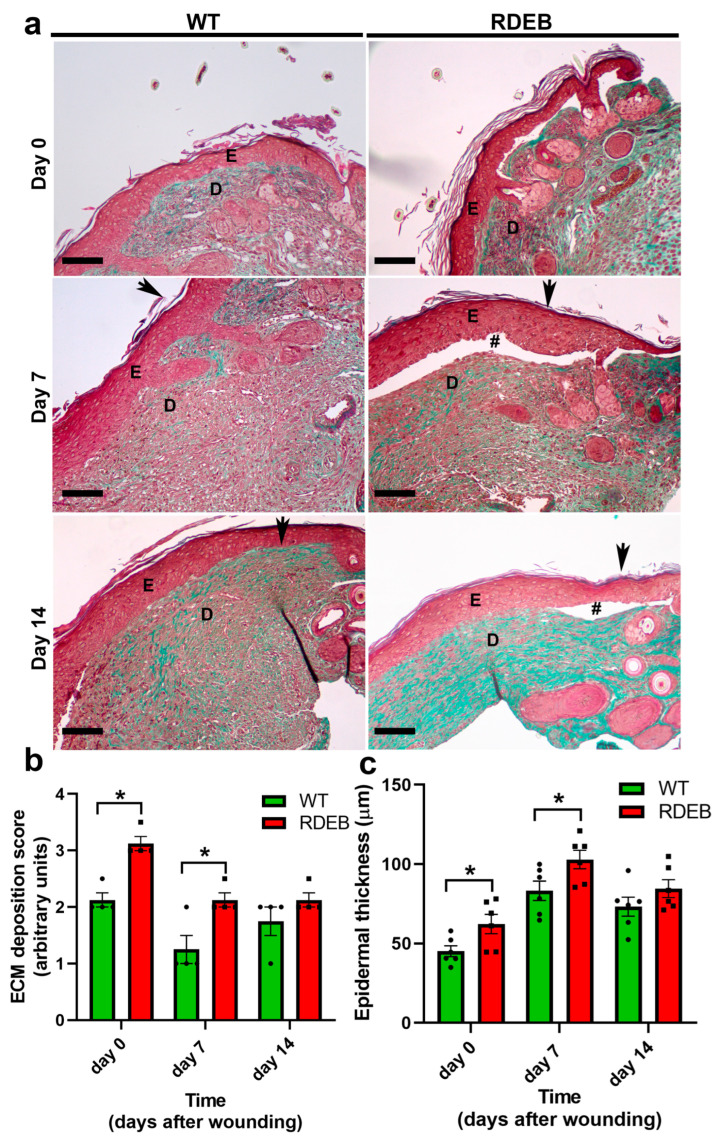
Increased Collagen I deposition and epidermal thickening in the paw skin of RDEB mice before and after wound induction. (**a**) Representative Masson’s trichrome-stained images of skin samples from WT and RDEB mice before wound induction (day 0) and at days 7 and 14 post-injury. E: epidermis; D: dermis; # indicates skin blistering, and arrows mark the wound border. Scale bar = 100 µm. (**b**,**c**) Quantification of extracellular matrix (ECM) deposition (**b**) and epidermal thickness (**c**). Data are presented as mean ± SEM (*n* = 4 per genotype). Asterisks indicate significant differences (* *p* < 0.05) determined by a two-tailed Mann–Whitney test.

Masson’s trichrome staining revealed an increase in overall extracellular matrix deposition in the RDEB animals before (day 0) and at 7 days post-wounding (Figure 3a,b). Interestingly, the RDEB animals also showed mild epidermal hyperplasia at days 0 and 7 (Figure 3a,c), a feature commonly observed in other inflammatory skin disorders such as psoriasis [21,22]. However, Masson’s trichrome staining does not distinguish between specific collagen types. To more precisely characterize the fibrotic alterations, we performed Picrosirius red staining under polarized light and an immunofluorescence analysis for Collagen I.

**Figure 4:**
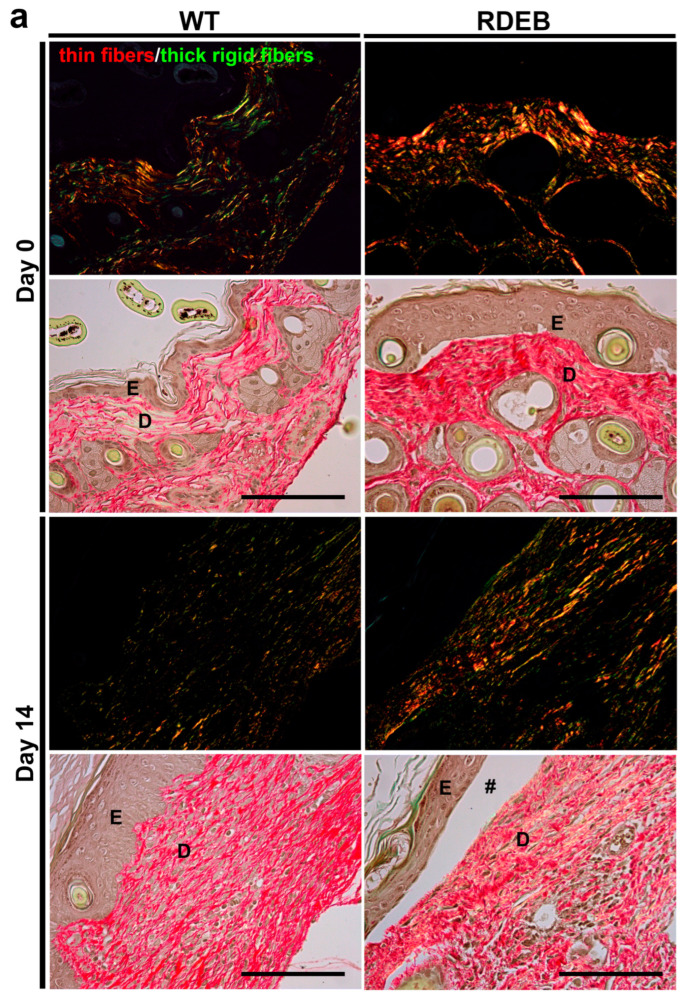

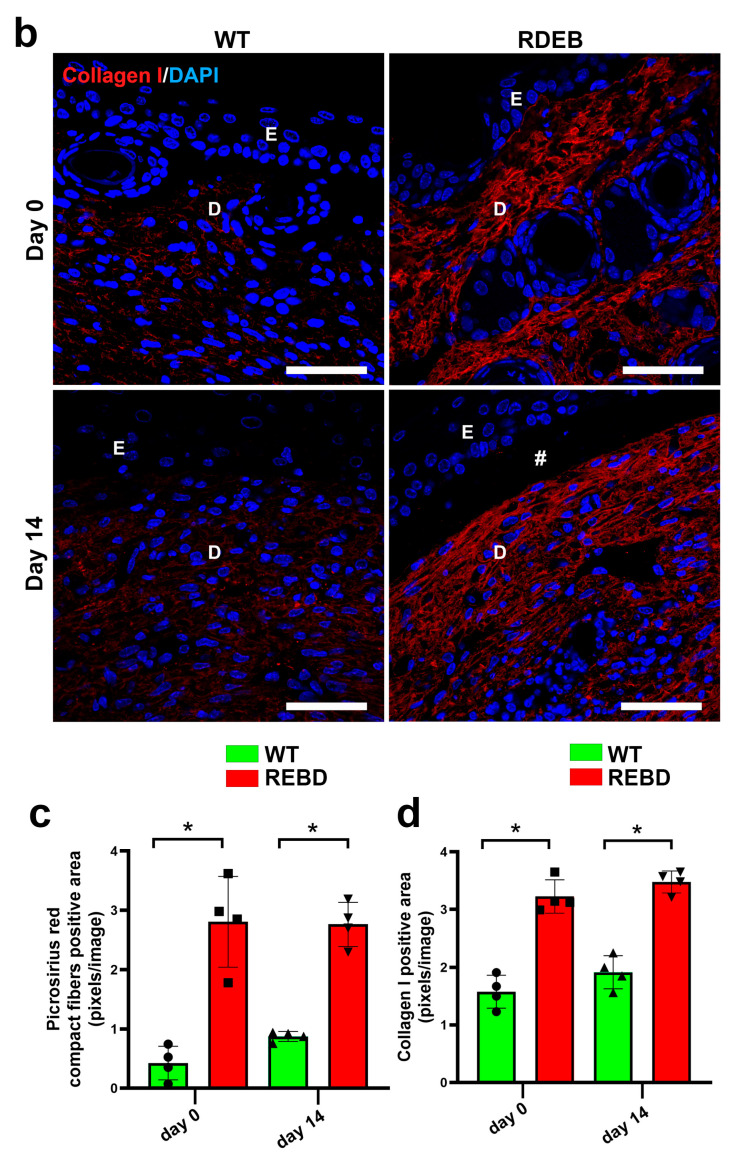
Altered collagen fiber organization and increased Collagen I deposition in the paw skin of RDEB mice before and after wound induction. (**a**) Representative images of Picrosirius red-stained sections viewed under polarized light (top panels) and bright-field light microscopy (bottom panels) in WT and RDEB mice before (day 0) and 14 days after injury. Under polarized light, thin and less organized collagen fibers appear green, whereas thick and highly crosslinked fibers appear orange-red. Notably, RDEB mice displayed a predominance of thick collagen fibers. E: epidermis; D: dermis; #: skin blistering. Scale bar = 100 µm. (**c**) Quantification of compact, birefringent collagen fibers (Picrosirius red staining under polarized light), expressed as positive area per image. (**b**) Representative confocal immunofluorescence images showing Collagen I (red) expression in WT and RDEB skin before (day 0) and 14 days after wound induction. Nuclei were counterstained with DAPI (blue). E: epidermis; D: dermis; #: skin blistering. Scale bar = 50 µm. (**d**) Quantification of Collagen I-positive areas (immunofluorescence staining). Bars represent mean ± SEM (*n* = 4 per genotype). Asterisks indicate significant differences (* *p* < 0.05), as determined by a two-tailed Mann–Whitney test.

Picrosirius red staining allowed us to differentiate thin (green) and thick (orange-red) collagen fibers. We observed that RDEB mice exhibited a predominance of thick, rigid collagen bundles both at baseline and 14 days post-injury (Figure 4a). Additionally, immunofluorescence staining for Collagen I demonstrated significantly increased expression in RDEB mice at both time points, confirming a sustained fibrotic response (Figure 4b). These findings were supported by quantitative analyses showing a greater area of Picrosirius red-positive thick fibers and higher levels of Collagen I immunoreactivity in the RDEB samples.

Importantly, the analysis was limited to day 0 (baseline) and day 14 (remodeling phase) to capture the initial fibrotic state of the tissue and the peak of matrix remodeling, respectively. These time points were chosen based on previous evidence showing that collagen production and reorganization are most active during the late healing phase, as well as to avoid redundancy with the Masson’s trichrome data already collected at days 0, 7, and 14.

Taken together, these results indicate that RDEB mice exhibit early and sustained histological abnormalities, including altered collagen architecture and fibrotic remodeling, which may contribute to delayed wound resolution.

### 2.3. C7-Hypomorphic Mice Exhibit Increased Inflammation and Exacerbated Fibrotic Markers in Paw Skin

**Figure 5:**
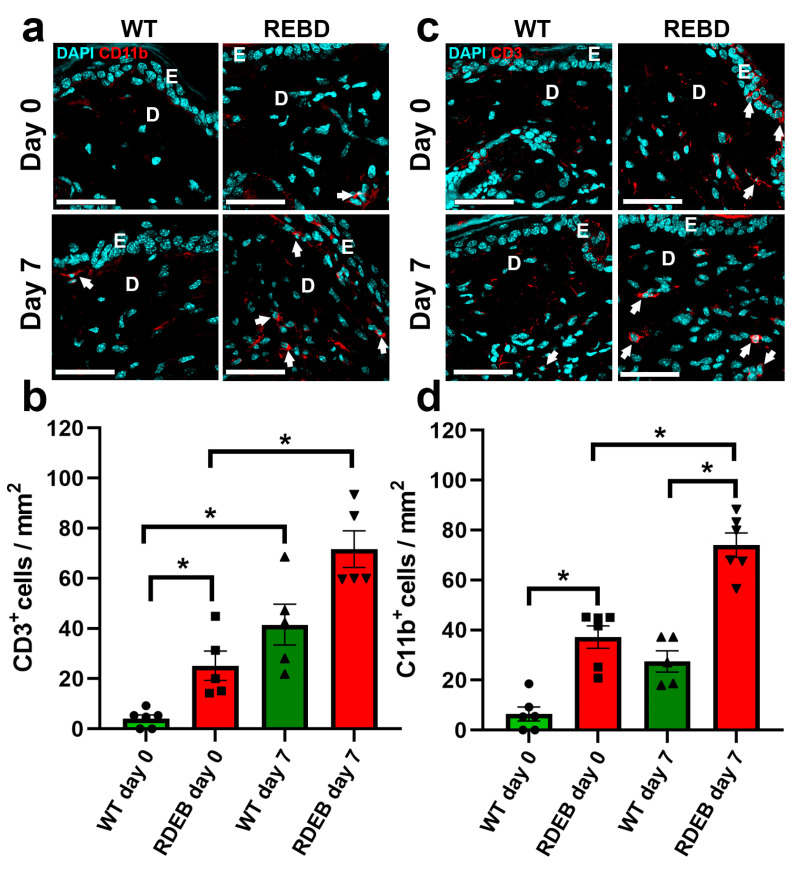
Enhanced inflammatory infiltration in the paw skin of RDEB mice before and after wound induction. (**a**,**c**) Representative confocal images showing inflammatory cell infiltrates as positive for macrophages (CD11b+) (**a**) and T cells (CD3+) (**c**) before wound induction (day 0) and at day 7 post-injury. Nuclei were counterstained with DAPI. E: epidermis; D: dermis. Arrows indicate CD11b+ and CD3+ cells. Scale bar = 50 µm. (**b**,**d**) Quantification of CD11b+ (**b**) and CD3+ (**d**) cell populations in skin samples. Data are presented as mean ± SEM (*n* = 5–6 per genotype). Asterisks indicate significant differences (* *p* < 0.05) determined by a Kruskal–Wallis test followed by Dunn’s post hoc test.

During wound healing, persistent inflammatory cell infiltration can impair tissue regeneration and contribute to the transition to chronic wounds [23]. To assess the inflammatory response in RDEB mice, we performed immunofluorescence staining for CD11b and CD3 markers, which identify macrophages and T cells, respectively, before wound induction (day 0) and seven days post-injury. Our results indicated that RDEB mice exhibit a slight but significant increase in macrophage (CD11b+) and T cells (CD3+) even under uninjured conditions, suggesting a pro-inflammatory state in intact skin. Additionally, CD11b+ cell infiltration was significantly higher at day 7 post-injury compared to the WT controls (Figure 5a–d), reinforcing the notion that RDEB wounds exhibit prolonged and heightened inflammatory responses.

To further characterize the innate immune response, we evaluated neutrophil infiltration by immunofluorescence using neutrophil elastase 2 (ELA2) as a specific marker. An analysis performed at day 7 post-injury, the time point of maximal inflammatory activity, revealed a significantly increased density of ELA2^+^ neutrophils in RDEB mice compared to wild-type littermates, as shown in Supplementary Figure S2. These results indicate that both innate and adaptive immune components are exacerbated in RDEB wounds during the acute inflammatory phase.

**Figure 6:**
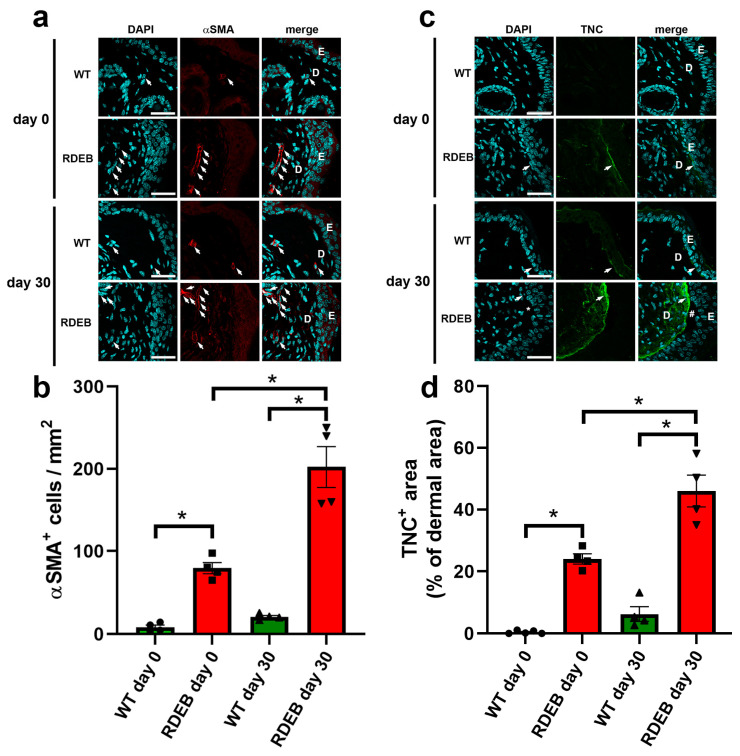
Fibrotic markers in the paw skin of RDEB mice before and after wound induction. (**a**,**c**) Representative confocal images showing myofibroblast (α-SMA) (**a**) and fibrotic scarring (tenascin-C [TNC]) (**c**) before wound induction (day 0) and 30 days post-injury. Nuclei were counterstained with DAPI. E: epidermis; D: dermis. Arrows indicate α-SMA-positive cells and TNC deposition. # indicates skin blistering. Scale bar = 50 µm. (**b**,**d**) Quantification of α-SMA-positive cells (**b**) and TNC-positive areas (**d**) in skin samples. Data are presented as mean ± SEM (*n* = 4–5 per genotype). Asterisks indicate significant differences (* *p* < 0.05) determined by a Kruskal–Wallis test followed by Dunn’s post hoc test.

Since the persistence of inflammatory infiltrate has been linked not only to a deficit in wound closure progression but also to fibrosis generation [24,25], we next examined fibrosis-associated markers before wound induction and at day 30 post-injury, when the wounds had fully re-epithelialized. α-SMA staining revealed a significant increase in myofibroblast density in the wound area both at 0 and 30 days in RDEB mice compared to the WT controls (Figure 6a,b). Additionally, we analyzed TNC, an extracellular matrix protein that is lowly expressed in healthy skin but highly upregulated after injury [26,27]. Interestingly, the RDEB mice displayed significantly higher levels of TNC in non-injured skin, with an even greater increase at day 30 post-injury (Figure 6c,d). Taken together, these results suggest that RDEB mice exhibit both basal and injury-induced inflammatory and fibrotic alterations, which may contribute to chronic wound development and excessive scarring.

### 2.4. C7-Hypomorphic Mice Exhibit Reduced Intraepidermal Nerve Fiber Density in Paw Skin

In addition to wound healing defects, RDEB is associated with severe pain, with over 50% of patients experiencing chronic pain on a daily basis [28,29]. Recent studies suggest that neuropathic pain in RDEB may result from damage to intraepidermal nerve fibers (IENFs), particularly at the distal ends of cutaneous nerves [30].

To evaluate nerve fiber alterations in this model, we performed an immunodetection of the PGP 9.5 antigen, a pan-neuronal marker, in hind paw cryosections before wound induction (day 0) and 30 days after injury, when the wound had fully healed and nerve fibers were undergoing reinnervation [31].

**Figure 7:**
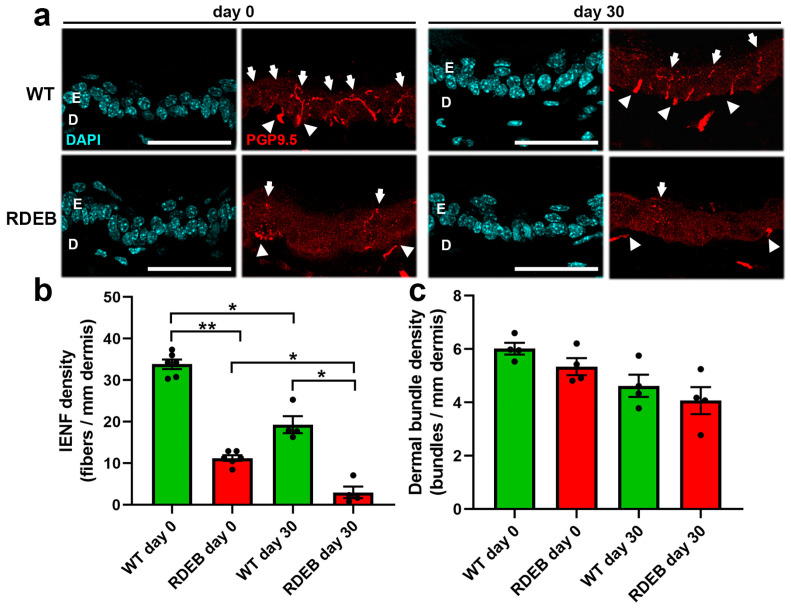
Reduced intraepidermal nerve fiber density in the paw skin of RDEB mice before and after wound induction. (**a**) Representative confocal images showing the intraepidermal nerve fibers (IENFs) in skin samples stained with the PGP 9.5 marker. Images are shown for samples collected before wound induction (day 0) and 30 days post-injury in WT and RDEB mice. E: epidermis; D: dermis. Arrows indicate IENFs, while arrowheads mark dermal nerve bundles reaching the epidermis. Scale bar = 100 µm. (**b**,**c**) Quantification of IENF density (**b**) and linear density of dermal nerve bundles (**c**). Data are presented as mean ± SEM (*n* = 4 per genotype). Asterisks indicate significant differences (* *p* < 0.05; ** *p* < 0.01) determined by a Kruskal–Wallis test followed by Dunn’s post hoc test.

At day 0, RDEB mice exhibited a significantly lower density of intraepidermal nerve fibers compared to WT mice (Figure 7a,b). Notably, nerve fiber regeneration after injury was also impaired in the RDEB mice, as evidenced by a lower IENF reinnervation rate at day 30 compared to the WT controls (WT: 57.8% ± 5.5; RDEB: 25.8% ± 6.6).

These findings, together with previous studies [30,32], suggest that the chronic pain experienced by RDEB patients, particularly in frequently damaged skin areas, may be due to both the loss of intraepidermal nerve fibers and their reduced regenerative capacity. Although the density of dermal nerve fiber bundles appeared slightly reduced in RDEB mice, no statistically significant differences were observed when compared to WT controls. These findings suggest that epidermal denervation in RDEB skin may not be solely attributed to a generalized loss of dermal innervation. However, the possibility of increased distal nerve degeneration in this model cannot be excluded (Figure 7c).

### 2.5. C7-Hypomorphic Mouse Paw Skin Exhibits Reduced Anchoring Fiber Density and Structural Alterations in the Dermal-Epidermal Junction After Injury

**Figure 8:**
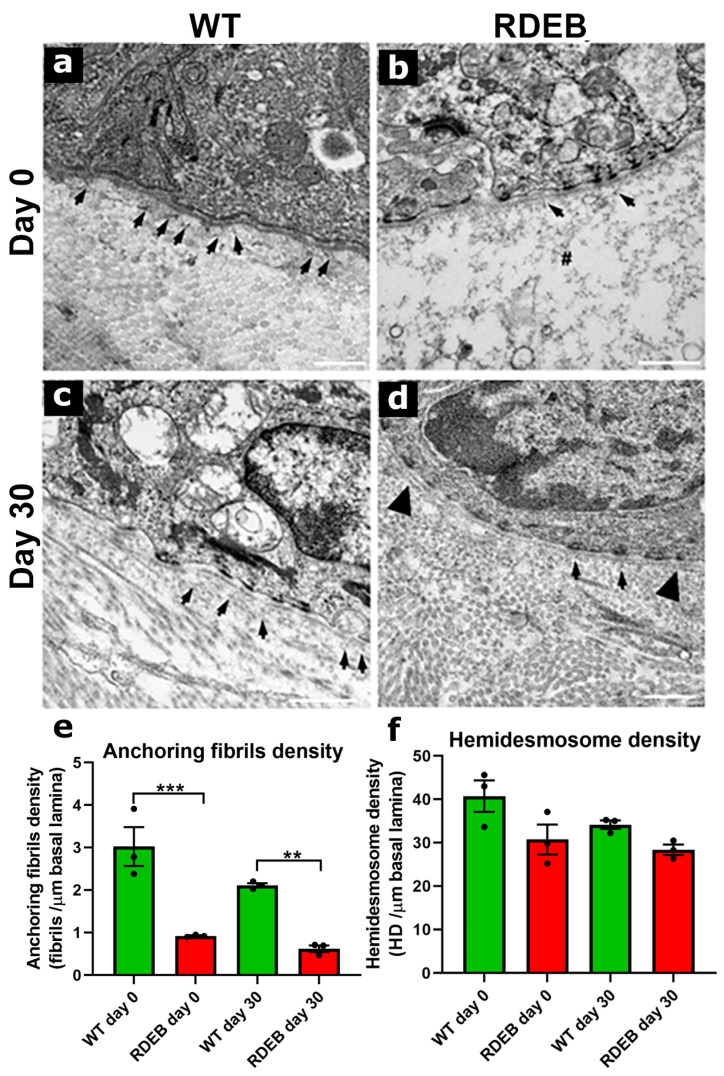
Reduced anchoring fibril density and structural alterations in the dermal-epidermal junction of RDEB mice. (**a**–**d**) Representative transmission electron microscopy (TEM) images showing the anchoring fibrils in skin samples collected before wound induction (day 0) and 30 days post-injury in WT and RDEB mice. Arrows indicate anchoring fibrils whereas # marks blistering sites, and arrowheads denote duplicated basal lamina regions. Scale bar = 2 µm. (**e**,**f**) Quantification of anchoring fibrils (**e**) and hemidesmosome linear density (**f**). Data are presented as mean  ±  SEM (*n* = 3 per genotype). Asterisks indicate significant differences (** *p* < 0.01, *** *p* < 0.001) determined by a Kruskal–Wallis test followed by Dunn’s post hoc test.

To investigate structural defects in the DEJ and anchoring fiber density, we performed a transmission electron microscopy (TEM) analysis on non-injured and post-injury (day 30) skin samples (Figure 8).

At low magnifications (6000–8500×), the data indicate that the WT and C7-hypomorphic mice did not exhibit major anatomical defects beyond the evident blister formation in the C7-hypomorphic mice (Supplementary Figure S2).

At higher magnifications (~20,000×), we quantified anchoring fiber density, which is critical for DEJ stability. RDEB mice exhibited a significantly reduced number of anchoring fibers and a thinner lamina densa compared to WT mice (Figure 8a,b,e), consistent with previous reports [9,16]. Other DEJ components, including the lamina lucida, hemidesmosomes, and collagen fibers, appeared similar between the groups (Figure 8, Supplementary Figure S3).

To assess anchoring fiber regeneration, we analyzed DEJ structure 30 days post-injury. The rate of anchoring fiber regeneration was comparable between the RDEB and WT mice (WT: 73.1% ± 6.8; RDEB: 68.2% ± 6.3) (Figure 8e). However, the RDEB mice exhibited structural abnormalities in the DEJ, including discontinuous basement membrane segments and duplicated lamina densa regions (Figure 8d). These aberrations have previously been associated with incomplete or defective tissue regeneration following injury [33,34].

These results confirm that RDEB mice exhibit significant structural defects in the DEJ, characterized by reduced anchoring fiber density, impaired regeneration, and architectural abnormalities following injury. Such alterations may contribute to chronic skin fragility and delayed wound healing in RDEB patients, reinforcing the relevance of this model for investigating new therapeutic strategies aimed at restoring DEJ integrity.

## 3. Discussion

### 3.1. Challenges in Developing a Comprehensive Therapy for RDEB

One of the major challenges in treating RDEB lies in the multifaceted nature of its pathological impairments, which have effects at both the systemic and local levels. Systemically, RDEB patients experience chronic inflammation; epithelial dysfunction, leading to nutritional deficiencies and respiratory complications; severe neuropathic pain; and various secondary manifestations. Locally, skin complications include delayed wound healing, mitten deformities, fibrotic scarring, and SCC development [10].

While systemic therapies such as gene therapy and cell-based treatments are currently under development, parallel efforts should be directed towards addressing the local challenges associated with difficult-to-heal wounds and SCC progression. The lack of suitable preclinical models for studying novel regenerative therapies or for testing anti-inflammatory and anti-fibrotic drugs further complicates the advancement of effective wound-healing strategies in RDEB.

### 3.2. Wound Healing Under Inflammatory and Fibrotic Conditions

Pro-inflammatory cytokines are produced as an early response to skin injury, regulating the functions of immune cells during the inflammatory phase and influencing keratinocyte and fibroblast activity during the proliferative phase. However, chronic inflammation, characterized by prolonged cytokine overexpression, is a hallmark of non-healing wounds in autoimmune and metabolic disorders [35,36,37] and has been directly correlated with RDEB severity [38].

Our findings align with these observations, as our RDEB mice exhibited increased inflammatory cell infiltration in the wound bed, which could contribute to delayed wound closure. However, the molecular mechanisms linking C7 deficiency to persistent inflammation remain unclear [38,39,40,41].

While inflammation and fibrosis are closely interconnected, extensive evidence from various animal models suggests that fibrosis significantly contributes to impaired wound healing [42,43,44,45]. In our study, we observed significantly elevated Collagen I deposition in healthy paw skin (before wound induction), suggesting that mechanical stress and high-friction environments may be sufficient to trigger fibrotic signaling in RDEB mice. Additionally, the increased myofibroblast presence and TNC deposition at 30 days post-injury suggest that fibrosis persists beyond the wound closure phase, further impairing tissue remodeling.

Finally, it is worth mentioning that in many models of impaired wound healing, vascular and angiogenic defects may be associated. Interestingly, the literature suggest that the regeneration defects in RDEB are not due to angiogenic deficiencies. In fact, several reports indicate an increase in angiogenic signaling in RDEB [46,47], which could be a factor stimulating the development of tumorigenic processes.

### 3.3. Limitations of the C7-Hypomorphic Mouse Model for Wound Healing Studies

One of the major limitations in using murine models for wound healing studies is the post-injury skin contraction process, which does not occur in humans [48]. This is due to the presence of a subcutaneous layer of striated muscle called the panniculus carnosus, which is largely absent in humans. In mice, this specialized muscle layer allows the independent movement of the skin from deeper muscles and is primarily responsible for rapid wound contraction following injury. To overcome this limitation, previous studies have validated the use of a silicone splint or ring, which is adhered to the wound edges to prevent contraction and promote epithelialization in a manner that mimics human wound healing [48,49,50]. However, the application of these devices is particularly complex in C7-hypomorphic mice due to their fragile and small skin structure, limiting their use in this model. Moreover, wound closure in the paw skin of mice does not undergo the contraction process observed in the back skin [51]. Instead, it follows a re-epithelialization pattern similar to that observed in human wounds, further reinforcing its value as a preclinical model for studying chronic wound healing in RDEB.

### 3.4. Difficult-to-Heal Wounds in RDEB: Future Perspectives

Currently, the lack of effective therapies for difficult-to-heal wounds represents a major public health concern and significantly impacts patients’ quality of life [52,53]. The scarcity of physiologically relevant animal models has hindered the development of novel therapeutic interventions for chronic, non-healing wounds [54].

This issue is particularly critical for RDEB patients, as their wounds exhibit persistent inflammation and fibrosis, especially in high-friction areas, leading to severe complications such as mitten deformities and SCC [10].

The primary aim of this study was to characterize a new preclinical model that recreates wound healing dynamics under pro-inflammatory and pro-fibrotic conditions. Our findings demonstrated that paw skin in RDEB mice exhibits significant structural and functional impairments even in the absence of wounding, including the following: (i) increased presence of inflammatory cells; (ii) excessive Collagen I accumulation; (iii) elevated α-SMA+ myofibroblast density; and (iv) aberrant tenascin-C expression.

These alterations worsened significantly upon injury, leading to defects in proliferation, migration, ECM remodeling, and sensory nerve localization. This last point is particularly relevant, as sensory nerve loss and dysfunction may be associated with neuropathic pain in RDEB patients, a poorly understood but clinically significant aspect of the disease [28,29,32].

Additionally, similar structural and functional abnormalities have been described in other inherited or acquired skin disorders. For instance, altered basement membrane composition and fibrillar matrix disorganization are also features of cutaneous aging and chronic UV exposure [33,34], and may contribute to impaired regeneration in other contexts of tissue fragility. Although poorly explored, recent reports have highlighted alterations in nerve fiber density and matrix remodeling in EB simplex and Ehlers–Danlos syndrome [38]. These findings support the notion that defective ECM architecture and innervation are shared pathological traits in genetic skin disorders, although their mechanistic underpinnings remain distinct.

In terms of anchoring fibril and basal membrane structure, our study demonstrates clear abnormalities in the dermal-epidermal junction (DEJ) of RDEB mice, even after re-epithelialization. These include reduced anchoring fibril density, duplicated lamina densa segments, and areas of basement membrane discontinuity, which may reflect defective repair processes. While anchoring fibril loss is a known feature of RDEB [30,55], our data provide new evidence of persistent ultrastructural defects following injury. To our knowledge, such post-injury architectural abnormalities in the DEJ have not been extensively described in human RDEB skin, representing a potentially novel observation with translational relevance.

### 3.5. Conclusions

Using paw skin as a preclinical model, we characterized a clinically relevant approach for studying fibrotic and inflammatory wound healing defects, offering a valuable platform for testing novel pro-regenerative, anti-inflammatory, and anti-fibrotic therapies. Given the clinical similarities between RDEB wounds and other chronic, difficult-to-heal skin conditions, this model may also be applicable beyond RDEB, serving as a powerful tool for developing targeted wound-healing therapies for fibrotic and inflammatory skin disorders.

## 4. Materials and Methods

### 4.1. Animals

The *Col7a1^flNeo/flNeo^* (C7-hypomorphic) mouse model was originally generated at the University of Freiburg (Germany) and was kindly provided by Dr. Alexander Nyström [16]. These mice express approximately 10% of wild-type type VII collagen (C7) and accurately recapitulate the clinical phenotype of RDEB, including delayed wound healing, skin fibrosis, and chronic inflammation [8,9,17].

Genotyping was performed using genomic DNA extracted from ear punches at postnatal day 7. PCR amplification was conducted using primers flanking the loxP insertion site in intron 2 of the *Col7a1* gene. The wild-type allele produced a ≈300 bp band, while the mutant *flNeo* allele generated a ≈500 bp band. PCR products were resolved on 2% agarose gels stained with ethidium bromide. Additionally, phenotypic screening was performed between postnatal day 1 and 30. Neonatal mice were inspected for signs of spontaneous skin fragility, including hemorrhagic erosions on the paws and tail. By one month of age, the mice were evaluated for mitten deformities and pseudosyndactyly formation in the forelimb and hindlimbs. Only animals that displayed both genotypic confirmation and consistent clinical phenotypes were included in this study. Representative images of genotypic and phenotypic features are provided in Supplementary Figure S4.

The mice were housed under controlled conditions (constant temperature, 50% humidity, and 12 h light/dark cycle) with ad libitum access to standard chow (LabDiet 5P00 RMH 3000, St. Louis, MO, USA) and water. To extend their lifespan, improve weight gain, and enhance overall health, the C7-hypomorphic mice remained with their mothers for four weeks until weaning, and the number of healthy littermates was reduced to minimize competition for breast milk. After weaning, the mice received daily nutritional supplementation, including grain-based infant cereal (Nestum, Nestlé, Vevey, Switzerland) and high-calorie dietary supplements (DietGel^®^ Boost, Clear H_2_O, Westbrook, ME, USA). Additionally, sunflower seeds were provided as an additional source of fat. Soft cellulose bedding was used to prevent spontaneous skin injuries. To minimize suffering, the C7-hypomorphic mice were euthanized at 13 weeks of age to prevent the onset of severe disease manifestations.

The mice were used at five weeks of age, which allowed a one-week acclimatization period after weaning at postnatal day 28, ensuring physiological stabilization prior to wound induction.

We designed our study to include an equal number of male and female animals per genotype during randomization, ensuring balanced representation and minimizing potential sex-related bias. This sex-balanced approach was informed by previous clinical studies and epidemiological reviews reporting no significant differences in RDEB incidence or phenotype between sexes [56,57]. Supplementary Figure S5 presents the sex-disaggregated analysis of body weight progression and wound closure kinetics, confirming that sex did not significantly affect these outcome measures in our model.

All procedures were approved by the Ethics Committee of Facultad de Medicina, Clínica Alemana-Universidad del Desarrollo (Protocol #02/2020), and complied with institutional and national animal welfare guidelines.

### 4.2. Wound Induction and Measurement of Ulcer Area

To establish a clinically relevant model of fibrotic wound healing, a full-thickness wound was induced in the paw skin, an anatomical site known for its high susceptibility to fibrosis and strong translational relevance to human RDEB patients [8,10].

At 8 weeks of age, the mice were anesthetized using sevoflurane vapors (Abbott, Tokyo, Japan). A 2.5 × 3.5 mm full-thickness excision wound was created on the dorsal surface of both hind paws using a scalpel, micro-dissecting scissors, and fine tweezers (Fine Sciences Tools, Foster City, CA, USA), following a previously established mouse foot ulcer model [58]. Post-surgical pain management was provided via tramadol (Laboratorio Chile, Santiago, Chile) administration for the first four days post-wounding. Digital images of the wounds were taken daily until complete epithelial closure, which occurred within 15–18 days. The wound area measurements (mm^2^) were quantified using ImageJ software, version 2.14.0 (NIH, Bethesda, MD, USA) [58]. The extent of wound closure was defined as the non-epithelialized area normalized to the initial wound size. Five to six mice per experimental group were used for each analysis.

### 4.3. Histological and Immunofluorescence Analysis of Wound Healing

The mice were euthanized via a ketamine (Troy Laboratories, Glendenning, NSW, Australia)/xylazine (Drag Pharma, Santiago, Chile) overdose. The wound area was excised, fixed in 4% paraformaldehyde, and then bisected through the center of the lesion to obtain the largest diameter of the wound. Histological assessment and immunofluorescence quantifications were independently analyzed in a blinded manner.

Tissue staining: For the determination of the epidermal area and collagen deposition, samples were paraffin-embedded, and 5 μm sections were stained with hematoxylin and eosin (H&E) and Masson’s trichrome, respectively. The inflammatory infiltrate and extracellular matrix deposition were quantified by a specialized pathologist in a blinded manner, using a severity score previously defined as 0, absence; 1, minimal; 2, moderate; 3, noticeable; and 4, markedly present [59]. The epidermal area and collagen deposition were quantified using ImageJ software, as previously described [60]. To further analyze the organization of collagen fibers, Picrosirius red staining was performed and visualized under polarized light, as previously described [17]. This method distinguishes thin (green) and thick (orange-red) collagen fibers, providing a semi-quantitative assessment of fiber maturity and crosslinking. Additionally, to specifically assess Collagen I expression, an immunofluorescence staining using anti-Collagen I antibodies was performed.

These two approaches were applied at day 0 (to define the baseline fibrotic state) and day 14 post-wounding, corresponding to the phase of extracellular matrix remodeling. These time points were selected to avoid redundancy with the Masson’s trichrome results and to capture critical changes in collagen dynamics during wound progression.

Immunofluorescence analysis: Fixed skin samples were immersed in 20% sucrose for 72 h and then embedded in Tissue-Tek O.C.T. (Sakura Finetek, Torrance, CA, USA). The skin samples were cryosectioned as 10 μm slices, blocked with 5% fish gelatin (Sigma Aldrich, St. Louis, MO, USA), and incubated overnight at 4 °C with the following primary antibodies (Supplementary Table S1): CD11b (macrophages/inflammatory infiltrates); CD3 (T cells/lymphocyte response); neutrophil elastase 2 (neutrophil infiltrate); α-SMA (myofibroblast/fibrosis); tenascin-C (ECM remodeling/fibrotic scarring); PGP 9.5 (sensory nerve fibers/neuropathy); and Collagen I (fibrotic matrix accumulation). After three washes with PBS, the samples were incubated with fluorescent secondary antibodies (Cell Signaling, Danvers, MA, USA) for 2 h at room temperature. Their nuclei were counterstained with DAPI (Applichem, Castellar del Vallès, Spain). A stack of 8–10 images per skin sample was obtained in a Fluoview FV10i confocal microscope (Olympus, Tokyo, Japan). For the quantification of intraepidermal nerve fiber density, the data were expressed as the number of PGP 9.5 positive fibers crossing from the dermis to epidermis per linear mm of skin [61]. Samples from 5 animals per experimental group were evaluated.

### 4.4. Transmission Electron Microscopy Analysis of the Dermo-Epidermal Junction

To assess anchoring fibril density and DEJ integrity, TEM was performed, as previously described [62]. Skin samples from wild-type (WT) and C7-hypomorphic mice were collected, fixed in 2.5% glutaraldehyde (Polysciences Inc., Warrington, PA, USA) in a 0.1 M cacodylate buffer (pH 7.0), and post-fixed in 1% osmium tetroxide. The samples were dehydrated, embedded in epoxy resin (Sigma-Aldrich, St. Louis, MO, USA), and ultrathin-sectioned (Ultracut R, Leica Microsystems, Vienna, Austria).

The sections were mounted on microscopy grids, stained with 5% uranyl acetate and 3% lead citrate (Sigma-Aldrich), and examined using a Talos F200C G2 transmission electron microscope (Thermo Fisher Scientific, Waltham, MA, USA). Anchoring fibril density was quantified using ImageJ software, focusing on randomly selected DEJ regions. Four mice per group were analyzed.

### 4.5. Statistical Analysis

All quantitative comparisons between two experimental groups were performed using a two-tailed Mann–Whitney test. For comparisons involving three or more groups, a Kruskal–Wallis test followed by Dunn’s post hoc test was applied. Quantitative data were presented as mean ± S.E.M., and statistical significance was set at *p* < 0.05. All statistical analyses were performed using GraphPad Prism software, version 8.4.3 (GraphPad Software, San Diego, CA, USA).

## Data Availability

The original contributions presented in this study are included in the article and Supplementary Materials. Further inquiries can be directed to the corresponding authors.

## Supplementary Materials

The following supporting information can be downloaded at: https://www.mdpi.com/article/10.3390/ijms26094281/s1.

## Abbreviations

The following abbreviations are used in this manuscript:

A-SMA	Alpha smooth muscle actin
C7	type VII collagen
DEJ	dermal-epidermal junction
EB	epidermolysis bullosa
ECM	extracellular matrix
IENFs	intraepidermal nerve fibers
RDEB	recessive dystrophic EB
SCC	squamous cell carcinoma
TEM	transmission electron microscopy
TGFβ	transforming growth factor beta 1
TNC	tenascin-C
