# Supplementary material for: Paw Skin as a Translational Model for Investigating Fibrotic and Inflammatory Wound Healing Defects in Recessive Dystrophic Epidermolysis Bullosa
Source: Int J Mol Sci. 2025 Apr 30;26(9):4281. doi: 10.3390/ijms26094281 (PMC12072301; doi:10.3390/ijms26094281)
Supplement: Supplementary file 1 [file ijms-26-04281-s001.zip › ijms-3565157-supplementary.pdf]

Supplementary Table S1. Summary of fluorescent stainings, primary and secondary antibodies for immunofluorescence.

**Primary antibody**	**#Cat. Number**	**Company**	**Dilution**	**RRID**
CD11b	14-0112-82	eBioscience	1:100	AB_467108
CD3	A0452	Dako	1:100	AB_2335677
ELA2	66972	Novus Biological	1:200	
α-SMA	MA5-11547	Invitrogen	1:200	AB_10979529
Tenascin-C	MAB2138	R&D	1:300	AB_2203818
Col1A1	#91144s	Cell Signaling	1:300	
PGP9.5	ab1761	Millipore	1:150	AB_2868444

**Secondary antibody**	**#Cat. Number**	**Company**	**Dilution**	**RRID**
α-mouse AF 488	A21202	Invitrogen	1:500	AB_141607
α-rabbit AF 555	4413S	Cell Signaling	1:500	AB_10694110
α-mouse AF 647	4410S	Cell Signaling	1:500	AB_1904023

**Stainings**	**#Cat. Number**	**Company**	**Dilution**	**RRID**
DAPI	4083S	Cell Signaling	1:200	-
Alexa 647-Phalloidin	A22287	Invitrogen	1:800	AB_2620155

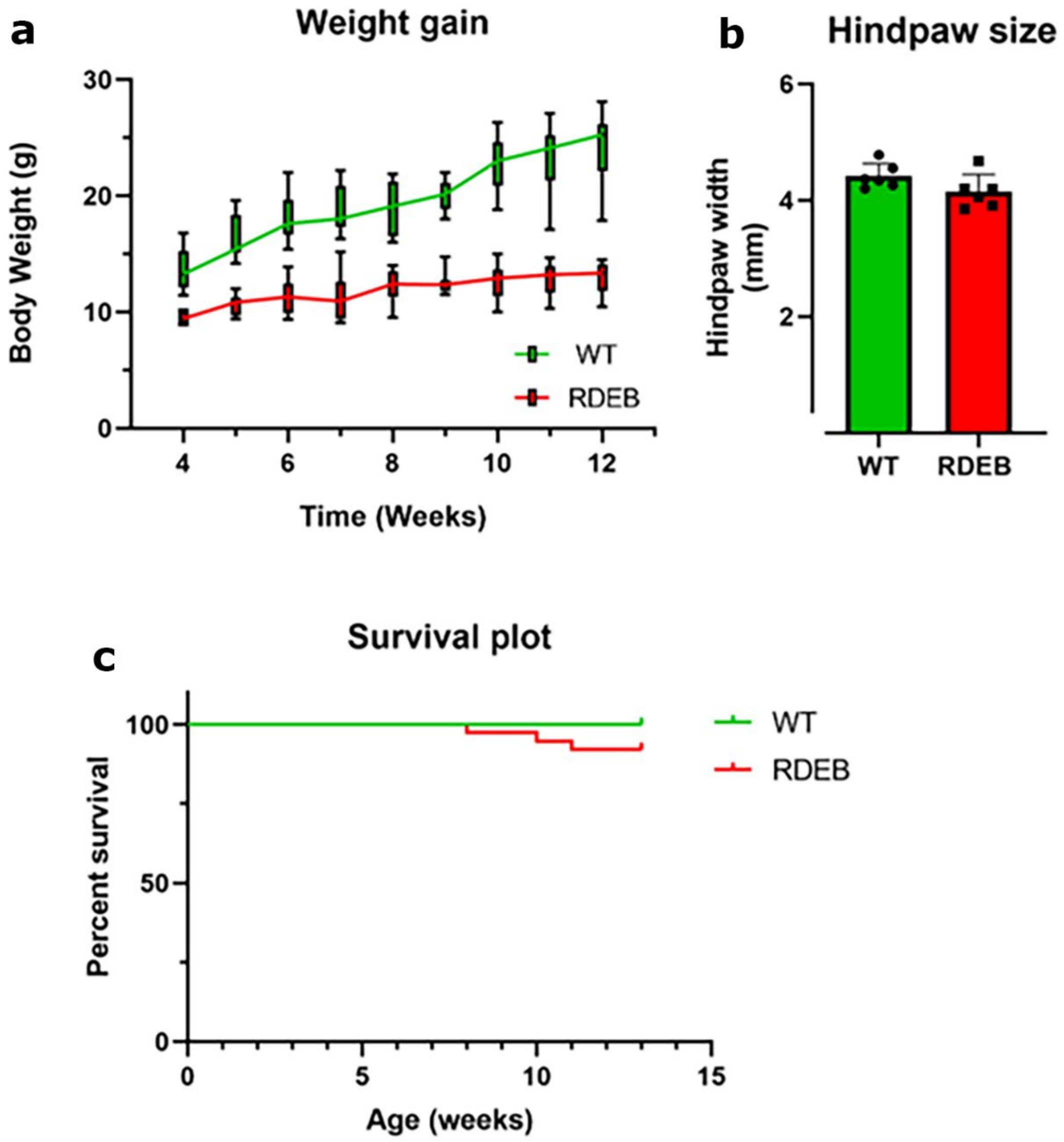

Supplementary Figure S1: Weight gain, hindpaw size and survival analysis in the RDEB mouse model.

(a) Analysis of body weight gain in WT and RDEB mice. No significant differences in weight gain were observed between males and females within each genotype (data not shown; n=22, 11 males and 11 females). **(b)** The hind paw width was measured in WT and RDEB mice at 13 weeks of age. No significant differences were observed between these animals (p=0.1; n=6). **(c)** Kaplan-Meier survival analysis in WT and RDEB mice. Notably, no major survival defects were evident in RDEB mice until the 13th week of life, when the animals were euthanized (p value = 0.214 vs WT mice, assessed by a Log-rank Mantel-Cox test). Data are presented as mean ± SEM, with a sample size of n=22 (11 males and 11 females) for each genotype.

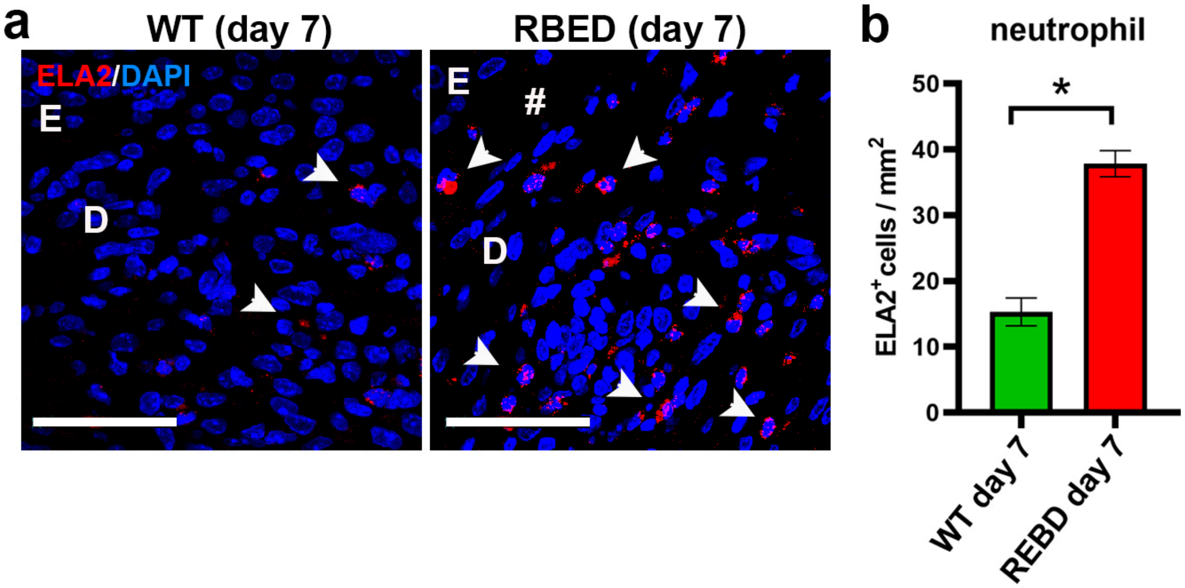

Supplementary Figure S2. Increased neutrophil infiltration in the paw skin of RDEB mice at day 7 post-injury. (a) Representative immunofluorescence images showing neutrophil elastase 2 (ELA2, red) and nuclear counterstaining with DAPI (blue) in paw skin cryosections from wild-type (WT) and RDEB mice at 7 days post-wounding. E: epidermis; D: dermis; # indicates skin blistering; arrowheads denote ELA2⁺ neutrophils. Scale bar = 100 µm. (b) Quantification of ELA2⁺ cells per mm² in WT and RDEB mice. Data are presented as mean ± SEM (n = 4 per genotype). Asterisks denote statistically significant differences (*$p < 0.05$) determined by a two-tailed Mann–Whitney test.

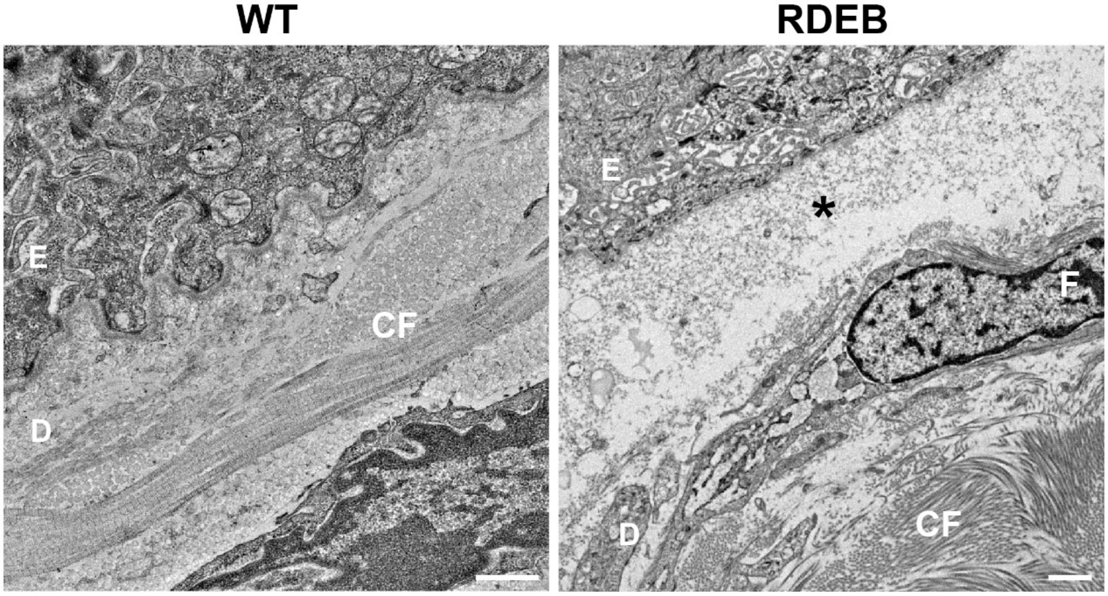

Supplementary Figure S3: RDEB mice showed extensive blistering in dermo-epidermal junctions.

Representative transmission electron microscopy images showing the general morphology of the dermo-epidermal junctions in RDEB and WT mice, in unwounded paw skin. Samples from RDEB mice showed abundant blistering, but other structures, such as the hemidesmosomes, collagen fibers and fibroblasts appear similar compared to WT mice (n=3). E: Epidermis. D: Dermis. CF: Collagen fibers. F: Fibroblast nucleus. The asterisk indicates a blister site. Bar: 1 µm.

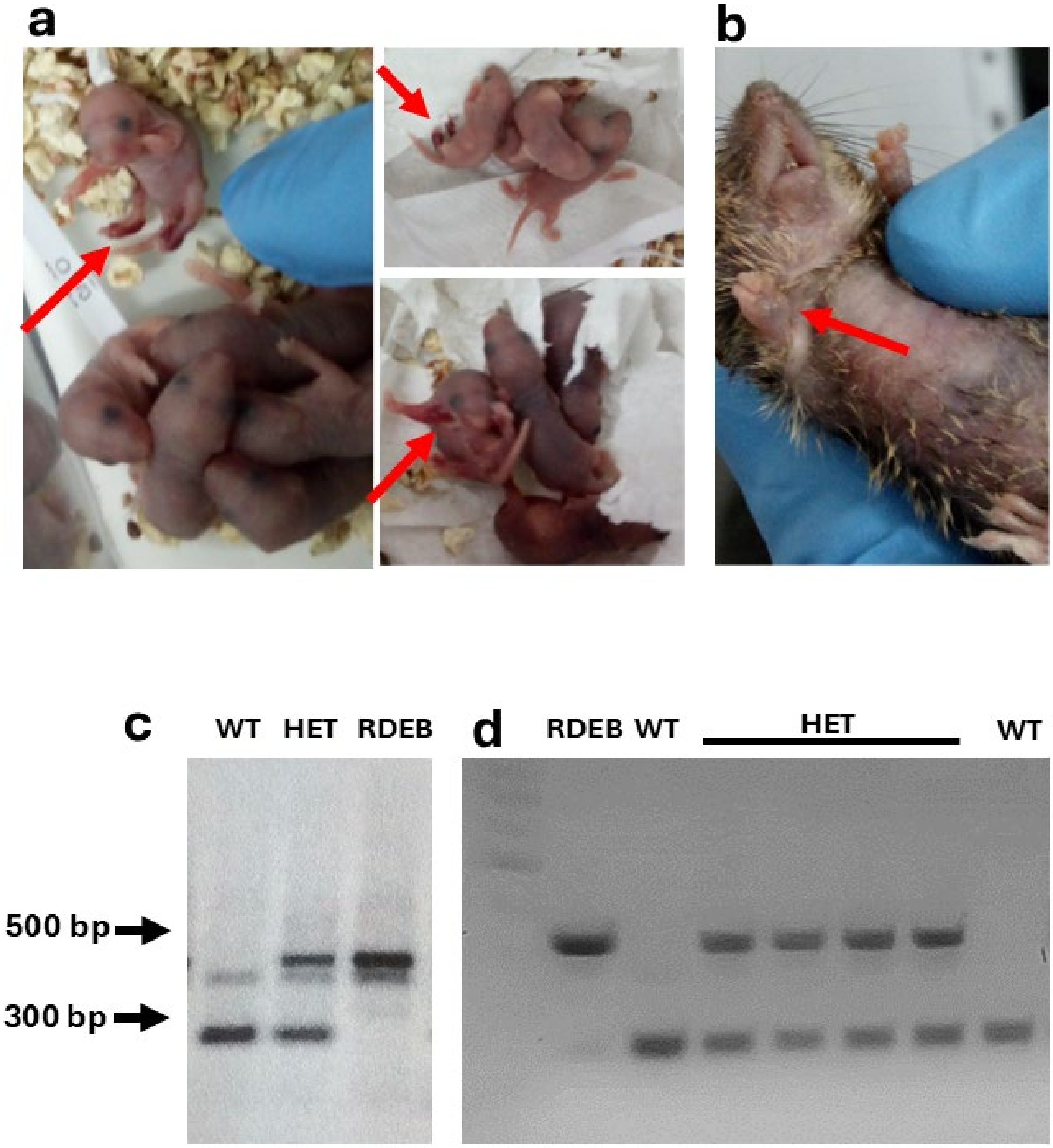

Supplementary Figure S4. Phenotypic and genotypic validation of Col7a1^flNeo/flNeo^ mice used in this study.

(a) Macroscopic appearance of neonatal RDEB (Col7a1^flNeo/flNeo^) mice showing spontaneous skin erosions on the paws within the first 48 hours after birth (red arrows). **(b)** Representative image of a one-month-old RDEB mouse exhibiting mitten deformities due to progressive pseudosyndactyly formation (red arrow). **(c)** Representative agarose gel image showing the genotyping pattern obtained by PCR amplification of genomic DNA from WT, heterozygous (HET), and homozygous RDEB (Col7a1^flNeo/flNeo^) mice. The wild-type allele produces a ≈ 300 bp band, while the mutant allele produces a ≈ 500 bp band. **(d)** Genotyping results from a representative litter confirming Mendelian segregation and identification of WT, HET, and RDEB animals. Only mice exhibiting both the phenotypic hallmarks and correct genotypic confirmation were included in the experimental procedures.

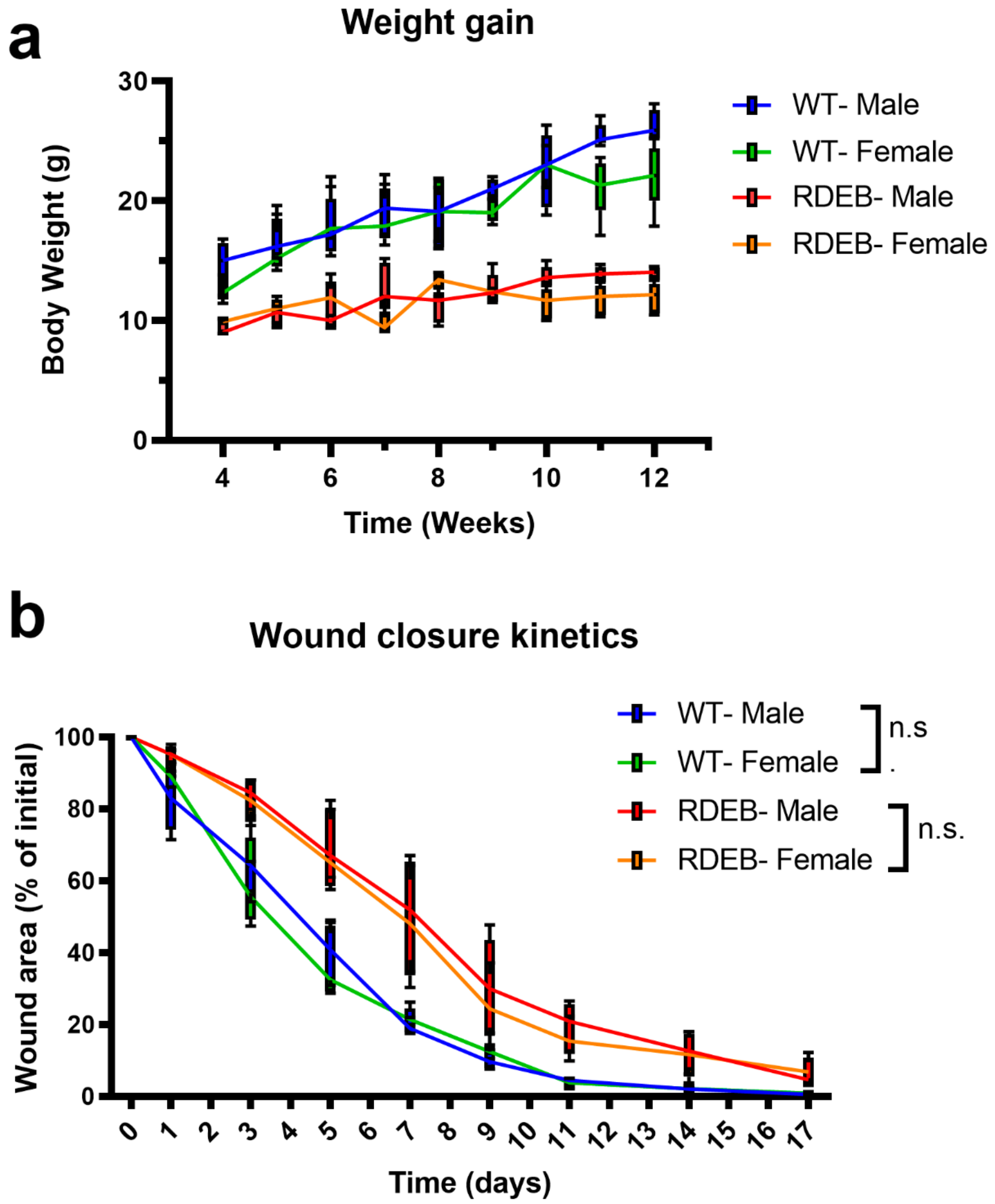

Supplementary Figure S5. Body weight and wound closure kinetics in WT and RDEB mice segregated by sex. (a) Weight gain curves of WT and RDEB mice (n = 4 per sex and genotype) from 4 to 12 weeks of age. Data are presented as mean ± SEM. Male WT animals displayed the highest weight gain, while RDEB mice of both sexes showed impaired growth. (b) Wound closure kinetics analyzed separately in male and female WT and RDEB mice. Data are shown as percentage of remaining wound area over time, normalized to day 0. No statistically significant differences were observed between sexes within each genotype. Data are presented as mean ± SEM. Statistical significance was assessed by a two-tailed Mann-Whitney test.
